# Graphical user interface for visualization of a decision support system for PEEP titration

**DOI:** 10.1186/cc13468

**Published:** 2014-03-17

**Authors:** S Lozano-Zahonero, S Buehler, S Schumann, J Guttmann

**Affiliations:** 1 University Medical Center Freiburg, Germany

## Introduction

The analysis of dynamic volume-dependent compliance provides the rationale basis for PEEP titration during mechanical ventilation. Due to its volume dependence, the compliance of the respiratory system is nonlinear within each single breath (intratidal), which is reflected in the compliance-volume curve (CV curve). The shape of the CV curve is determined by the PEEP level. The mechanical stress for the mechanically ventilated lung is expected to be minimal when the lung is ventilated within the volume range of maximal compliance. We present a new graphical user interface (GUI) that supports the clinician to titrate the PEEP level by means of a shape category analysis of the CV curve.

## Methods

A decision support system in the form of a computer-based GUI was developed and tested using respiratory data obtained from patients of the University Medical Center Freiburg. The new clinician- oriented approach provides a breath-by-breath visualization of the patient's individual intratidal CV curve. The dynamic compliance was analyzed using the gliding-SLICE method [1]. The resulting intratidal CV curve was classified into one of six shape categories according to the definition from Mols and colleagues [2]. The actual shape category of the CV curve indicates whether PEEP setting should be changed or whether the volume range of maximal compliance is reached.

## Results

The GUI provided a breath-by-breath visualization of the intratidal CV curve and the intuitive individual compliance shape category. Based on the compliance shape category, different guidelines of PEEP titration were applied with the objective of ventilating the patient mechanically within the range of maximal compliance.

## Conclusion

The newly developed GUI allows a breath-by-breath visualization of the intratidal CV curve with high reliability. Automated classification of the intratidal CV curve into compliance shape categories provides the rationale basis for patient-individual PEEP titration.
